# Clinical Implications of Molecular and Genetic Biomarkers in Cushing’s Disease: A Literature Review

**DOI:** 10.3390/jcm14093000

**Published:** 2025-04-26

**Authors:** Laura Chinezu, Maximilian Cosma Gliga, Mihnea Bogdan Borz, Camelia Gliga, Ionela Maria Pascanu

**Affiliations:** 1 Department of Histology, George Emil Palade University of Medicine, Pharmacy, Sciences and Technology of Targu Mures, 540142 Targu Mures, Romania; laura.chinezu@umfst.ro (L.C.); camelia.gliga@umfst.ro (C.G.); 2 Department of Endocrinology, George Emil Palade University of Medicine, Pharmacy, Sciences and Technology of Targu Mures, 540142 Targu Mures, Romania; ionela.pascanu@umfst.ro; 3 Department of Anatomy, George Emil Palade University of Medicine, Pharmacy, Sciences and Technology of Targu Mures, 540142 Targu Mures, Romania; bogdan.borz@umfst.ro

**Keywords:** Cushing, pituitary, corticotropinoma, biomarkers, molecular

## Abstract

Cushing’s disease (CD) is a rare disorder caused by adrenocorticotropic hormone (ACTH)-secreting pituitary neuroendocrine tumors, which lead to chronic hypercortisolism and significant complications with increased mortality. These tumors are characterized by a substantial heterogeneity in their biological behavior, prognosis, and therapeutic response, making their management challenging. While transsphenoidal surgery remains the first-line treatment, recurrence rates remain high, and alternative therapeutic approaches, such as pharmacological therapy and radiotherapy, have a variable efficacy and are frequently limited due to side effects. Increasing evidence suggests that molecular biomarkers, both immunohistochemical and genetic, may play an important role in predicting a tumor’s aggressiveness, recurrence risk, and response to targeted therapies. The immunohistochemical evaluation of its granulation pattern, Ki-67 proliferation index, and E-cadherin expressions have been linked to a tumor’s invasiveness and surgical outcomes, while somatostatin and dopamine receptor expressions may influence its response to Pasireotide and cabergoline therapy. Genetic alterations such as USP8 mutations impact tumor growth and its response to targeted therapies, whereas CABLES1 and TP53 alterations may contribute to more aggressive tumor behavior. Despite these findings, the clinical applicability of many of these markers remains limited by inconsistent validation and lack of standardized cutoff values. This narrative review provides an update on the latest evidence regarding the roles of molecular biomarkers in corticotropinomas, emphasizing their role in prognosis, recurrence risk, and the response to different treatment options. A better understanding and integration of these biomarkers into clinical practice could lead to a better patient stratification, more efficient therapeutic strategies, and personalized treatment approaches for patients with CD.

## 1. Introduction

Cushing’s disease (CD) is a chronic endocrine disorder caused by a corticotroph pituitary neuroendocrine tumor (PitNET), leading to excess ACTH and cortisol production. This results in metabolic abnormalities, including impaired glucose metabolism, a risk of type 2 diabetes, cardiovascular complications, and increased mortality if untreated [1,2]. CD is rare, with an incidence of 0.24 cases per 100,000 person/year and a prevalence of 2.2 per 100,000. It affects females more often, with a female-to-male ratio of 3:1 to 15:1, and it is typically diagnosed between 25 and 40 years of age [3,4]. Despite diagnostic advances, delayed detection remains common, increasing morbidity and mortality.

Transsphenoidal surgery is the first-line treatment, with remission rates of 70–80%, though recurrence occurs in 30% of cases, particularly in larger, invasive tumors [1,5,6]. For persistent disease, second-line options include reintervention, radiotherapy, and pharmacological treatment, though radiotherapy carries risks of hypopituitarism. Pasireotide, a somatostatin agonist, reduces ACTH and cortisol by targeting SSTR5 receptors, but it may cause hyperglycemia and has a variable efficacy [7,8,9]. Emerging therapies, such as USP8 inhibitors and mifepristone, show promise but require further validation [1,10,11,12].

Corticotropinomas exhibit clinical, pathological, and molecular heterogeneity. While most are slow-growing and well differentiated, up to 20% are aggressive, characterized by rapid growth, invasiveness, and recurrence after surgery. Subtypes like Crooke’s cell tumors and silent corticotroph tumors complicate diagnosis and treatment [13,14,15]. Molecular alterations, including changes in cell signaling and epigenetics, are emerging as biomarkers to predict tumor behavior and prognosis [4,16,17].

A milestone in understanding corticotropinomas was the 2022 WHO classification, which redefined pituitary tumors as neuroendocrine neoplasms. This classification emphasizes subtyping based on TPIT expression and stratifies corticotropinomas into densely and sparsely granulated tumors and Crooke’s cell adenomas, each with a distinct behavior and clinical implications [14,18]

Recent research into molecular and genetic factors has enhanced our understanding of corticotropinoma pathogenesis, identifying biomarkers that influence tumor behavior, therapy response, and prognosis. USP8 mutations, present in about 35–60% of corticotroph tumors, increase EGFR signaling, promoting ACTH secretion and growth, yet are linked to smaller, less invasive tumors and a better response to Pasireotide. In contrast, TP53 mutations, though rarer, are associated with aggressiveness, invasiveness, and a higher recurrence risk [4,17,19,20,21]. Other alterations, such as USP48 mutations, may contribute to progression and therapy resistance. Epigenetic changes, including altered microRNA expression and DNA methylation, are also under investigation for roles in progression and resistance [4,17,19].

Immunohistochemical (IHC) biomarkers like Ki-67, p53, and E-cadherin correlate with tumor aggressiveness; a high Ki-67 and low E-cadherin indicate greater proliferation and invasiveness. SSTR5, Pasireotide’s target, can be assessed via IHC, and its absence may signal resistance [4,22,23,24]. Integrating these biomarkers into clinical practice may support personalized treatments, improving outcomes and reducing morbidity and mortality.

## 2. Aim and Methods

This review aims to explore the role of molecular and genetic biomarkers in corticotropinomas, with a focus on their impact on tumor classification, clinical behavior, invasiveness, and its response to different therapeutic strategies. Through this article we have tried to summarize current knowledge on both genetic and immunohistochemical biomarkers relevant to tumor behavior and treatment response, assessing their potential role in guiding personalized management approaches in CD. A narrative review was conducted through a literature search in PubMed, Scopus, and Web of Science, using keywords such as Cushing’s disease, corticotroph tumors, corticotropinoma, genetic features, molecular, and immunohistochemistry. Articles were selected based on their relevance to our subject, with a particular focus on research published in the last 10 years. Additional references were obtained from recent published reviews, meta-analyses, and expert guidelines. Finally, we synthetized our findings to provide an overview of the latest knowledge on molecular biomarkers and how these contribute to tumor characterization, disease progression, and treatment response, with a focus on their clinical significance.

## 3. Genetic Mutations

### 3.1. USP8 Mutations

The discovery of the USP8 gene has been a major discovery in understanding the molecular mechanisms behind CD, as these mutations are widely present in corticotropinomas, with their prevalence estimated at around 35–60% of cases [25,26,27]. This gene encodes a deubiquitinase enzyme responsible for regulating the degradation of epidermal growth factor receptor (EGFR), an important factor in cell growth and hormone production. In normal conditions, EGFR undergoes degradation, which helps regulate its signaling activity, but mutations in the exon 14 of USP8 disrupt this process by activating the enzyme, leading to prolonged EGFR signaling, increased ACTH secretion, and, ultimately, cortisol excess, which leads to the clinical syndrome of CD [28].

The reported prevalence of somatic USP8 variants in corticotropinomas shows a substantial variation, ranging from 11% to 65% across published cohorts. This heterogeneity likely arises from several factors. Methodological discrepancies in mutation detection could play a significant role; next-generation sequencing (NGS) has demonstrated higher sensitivity compared to Sanger sequencing, especially in samples with a low tumor cellularity or formalin-fixed paraffin-embedded tissues. Discrepancies can also occur due to differences in patient selection criteria; tumor classification, such as the inclusion of silent corticotropinomas; and the ethnic or geographic origin of cohorts. Asian studies, such as those by Ma et al. [27] and Shichi et al. [29], reported a higher prevalence (62% and 65%, respectively), whereas European and Brazilian studies reported lower frequencies (23–43%) [30]. We consider that these differences highlight the influence of both a technical and population-based heterogeneity in prevalence estimates, and a harmonized diagnostic approach and multicenter, multiethnic validation studies are essential to accurately define the true prevalence and clinical implications of USP8 mutations.

The clinical relevance of USP8 mutations has been studied extensively in recent research on genetic mutations in corticotropinomas. Several authors consistently report that USP8 mutations are more frequent in female patients, with the reported prevalence among women ranging from 27% to 68% across cohorts [30]. A study by Perez-Rivas et al., conducted on 145 ACTH-producing pituitary tumors, found that USP8 mutations were significantly more frequent in females (43%) compared to males (17%) and were associated with an earlier age of diagnosis [31]. Reincke et al. [32] found USP8 mutations exclusively in females (100% vs. 45% in wild-type), and Ma et al. [27] observed a higher frequency in females (67%) than in males (38%). However, this gender association is not uniformly observed, as other authors have found no significant sex-based difference [30]. Given that CD itself occurs 3–4 times more frequently in women, it remains unclear whether the higher USP8 mutation frequency in females is a causal factor in this epidemiological trend or simply a reflection of the overall sex bias in the disease’s incidence. It seems plausible that USP8 mutations may define a specific molecular subtype of CD that is more common among women, though mechanistic studies are still lacking. Further research is needed to determine whether sex-specific genetic, epigenetic, or hormonal factors drive the emergence of USP8-mutant tumors, and whether this contributes directly to the gender disparity in corticotropinomas’ incidence.

Several studies suggested that USP8-mutant tumors are smaller and less invasive, often making them more likely to achieve complete surgical resection. Additionally, patients with USP8-mutant tumors seem to experience better postoperative outcomes, with higher remission rates compared to those with wild-type tumors [27,33]. In the study by Hayashi et al. on 60 corticotroph tumors, it was found that USP8-mutant tumors were more frequently microadenomas (<10 mm) and were less likely to invade surrounding structures, particularly the cavernous sinus [34]. These findings were also confirmed in another study by Perez-Rivas et al., which reported that USP8-mutant tumors had a median size of 10 × 7 mm and were significantly less likely to extend beyond the sella turcica [31]. However, the same study found conflicting evidence, suggesting that USP8-mutated adenomas were less likely to result in a postoperative adrenal insufficiency and also presented significantly higher post-operative urinary free cortisol levels, suggesting a potentially higher risk of late-recurrence after surgery or persistent disease [31]. Furthermore, another recently published study by Locantore et al. [33] found that USP8-mutated ACTH-secreting tumors were found to be predominantly microadenomas, with significantly lower rates of cavernous sinus invasion compared to wild-type tumors. In the same study, patients with USP8 mutations had higher remission rates following transsphenoidal surgery, suggesting a more favorable initial surgical outcome. However, the lower incidence of postoperative adrenal insufficiency found in patients with this mutation raises concerns about potential late recurrence, likely due to residual tumor activity [33]. Interestingly, a study by Sesta et al. [26] from 2020 found that USP8-mutated ACTH-secreting pituitary tumors have a distinct molecular and functional profile compared to wild-type tumors. In this study, the authors identified USP8 mutations in 23% of analyzed tumors and observed that these tumors produced higher levels of ACTH and had an increased POMC expression in vitro, suggesting that, despite being smaller in size, they are highly active in hormonal secretion. The study also showed that USP8-mutated tumors were more responsive to corticotropin-releasing hormone (CRH) stimulation and dexamethasone suppression, indicating that they maintain a more typical corticotroph function in terms of hormonal regulation [26].

Beyond their implications for tumor behavior and invasiveness, recent studies found that USP8 mutations might also have implications for pharmacologic treatment responsiveness [34]. There is growing evidence that USP8-mutant corticotropinomas express higher levels of STTR5, which may explain their increased sensitivity to somatostatin receptor ligands like Pasireotide. A study by Castellnou et al. [20] explored the relationship between USP8 mutations and SSTR5 expression in corticotroph tumors, along with their potential influence on the responsiveness to Pasireotide. Among the 50 tumors, USP8 mutations were found in 26%, with a significantly higher prevalence in functioning corticotroph tumors compared to silent ones. SSTR5 expression was more frequent in USP8-mutated tumors than in wild-type tumors (*p* = 0.007), suggesting a possible link between USP8-driven molecular alterations and SSTR5 upregulation [20]. These findings were also observed in the study by Hayashi et al. [34], where USP8-mutated tumors showed a significantly higher expression of SSTR5. Additionally, non-mutant aggressive tumors exhibited a low expression of Methylguanine-DNA–methyltransferase (MGMT), which is typically associated with responsiveness to temozolomide, indicating that USP8 wild-type tumors may benefit more from alkylating chemotherapy than from somatostatin analogs [34]. Furthermore, the study by Albani et al. [35] also confirmed that USP8-mutated corticotroph tumors exhibited a higher expression of SSTR5, correlating with an improved response to Pasireotide [35]. In vitro, Pasireotide more effectively suppressed ACTH secretion in USP8-mutant tumors compared to the wild-type, likely due to enhanced SSTR5 transcription. Interestingly, a recent study by Treppiedi et al. [36] found that not all USP8 mutations confer the same sensitivity to Pasireotide. Specifically, while most USP8 mutations did not enhance responsiveness to Pasireotide, the P720R mutation was associated with a significantly better response to Pasireotide, leading to greater ACTH suppression [36]. All these findings suggest that USP8 mutational status could serve as a predictive biomarker for Pasireotide responsiveness in Cushing’s disease, though further clinical studies on patients treated with Pasireotide are needed to confirm this association.

Furthermore, USP8 inhibitors are emerging as a promising therapeutic option for CD. Preclinical studies, like the one performed by Kageyama et al. [10], have shown that targeting USP8 with specific inhibitors can suppress ACTH production, reduce tumor cell proliferation, and induce apoptosis in corticotroph tumor models. These findings highlight USP8 as a promising therapeutic target for USP8-mutated corticotroph tumors. Although these inhibitors are not yet available for clinical use, in the future they might hold great potential for patients with persistent or recurrent CD who do not respond well to standard therapies [10].

### 3.2. USP48 Mutations

The ubiquitin-specific protease 48 (USP48) gene was proposed as another important factor in the pathogenesis of USP8-wild-type corticotroph tumors. Similar to USP8, USP48 encodes a deubiquitinase involved in protein degradation pathways. In a study by Chen et al. [37], recurrent USP48 mutations were identified in approximately 23% of USP8-wild-type corticotroph tumors. These mutations were mutually exclusive with USP8 and BRAF mutations, highlighting USP48 as an independent driver of tumorigenesis in a subset of CD patients. From a functional point of view, USP48 mutations were shown to enhance POMC promoter activity and transcription, thus contributing to increased ACTH production, similar to the effects observed with USP8 mutations [37,38]. Further research on this gene was conducted in a study by Sbiera et al. [39], which found that USP48 mutations were present in about 10% of corticotroph tumors, with a trend toward a higher frequency in female patients [39]. Furthermore, it was found that tumors harboring USP48 mutations were associated with a smaller tumor size, often presenting as microadenomas. Functionally, USP48 mutations were associated with the increased responsiveness of corticotroph tumors to CRH stimulation, leading to increased ACTH secretion, supporting the role of this mutation in the pathogenesis of CD [39]. Collectively, the available data suggest that USP48 mutations contribute to CD’s pathogenesis and are a common mutation in USP8-wild-type CD, associated with a smaller tumor size and a tendency towards the female gender. However, further studies are needed to fully elucidate the clinical implications of USP48 mutations, including their potential role in predicting treatment response or tumor recurrence. Future research might also focus on the discovery of the potential of USP48 as a therapeutic target, particularly in USP8-wild-type CD tumors where conventional treatment options may be less effective.

### 3.3. X-Linked Ubiquitin-Specific Peptidase 11 (USP11)

Recent studies have identified the X-linked deubiquitinating enzyme USP11 as a novel susceptibility factor in the pathogenesis of CD, particularly contributing to its higher prevalence among females. Elevated expression of USP11 has been observed in females with ACTH-secreting PitNETs when compared to male counterparts or normal pituitary tissue. Mechanistically, USP11 directly interacts with and deubiquitinates the transcription factor TPIT, a key regulator of POMC, the precursor of ACTH. This deubiquitination stabilizes TPIT, enhances POMC transcription, and ultimately promotes excessive ACTH secretion. The knockdown of USP11 in both in vitro cell lines and in vivo mouse models leads to reduced POMC expression and ACTH levels, confirming its functional role in tumor behavior. Notably, virtual screening approaches have identified Lomitapide and Nicergoline as potential USP11 inhibitors that suppress TPIT and POMC expression, decrease ACTH secretion, and exert antitumor effects without notable cytotoxicity [40]. These findings suggest that USP11 not only contributes to the gender bias observed in CD through mechanisms potentially linked to X chromosome inactivation escape but also presents a promising therapeutic target for novel treatments.

### 3.4. BRAF Mutations

The BRAF gene, well known for its role in oncogenesis via the activation of the MAPK/ERK signaling pathway, has also been implicated in the pathogenesis of pituitary corticotroph tumors, although at a much lower frequency compared to USP8 or USP48 mutations. In the study by Chen et al. [37], BRAF mutations were detected in approximately 16% of USP8-wild-type corticotroph adenomas, primarily involving the p.V600E hotspot. The authors demonstrated that BRAF-mutated tumors exhibited enhanced MAPK/ERK signaling, leading to increased activation of the POMC promoter and upregulated ACTH production. These findings suggest that BRAF-mutant tumors share a common pathogenic mechanism with USP8- and USP48-mutated tumors, in that all three promote corticotroph tumor hypersecretion, though via distinct upstream pathways [37]. Other studies have revealed notable geographic and ethnic variations in the prevalence of BRAF mutations. For example, a study by Sbiera et al. reported that BRAF mutations were absent in a European CD cohort, suggesting a much lower prevalence outside of East Asian populations [39]. Furthermore, a more recent prospective study by Abraham et al., involving a cohort from India, found no BRAF mutations in 29 USP8-wild-type corticotroph tumors, despite identifying USP48 mutations in a subset of these cases [41]. This discrepancy may reflect population-specific genetic backgrounds influencing the mutational landscape of corticotroph tumors. Clinically, while BRAF-mutated tumors have been associated with increased POMC transcription and ACTH secretion, there is currently limited data on their phenotype, including tumor size, invasiveness, or treatment response, due to the rarity of these mutations. While the therapeutic implications remain largely unknown, the identification of BRAF mutations in corticotroph tumors suggests that MAPK/ERK pathway inhibitors, such as BRAF inhibitors, might represent a future treatment option for selected cases that possess the mutation. However, additional studies are needed to validate BRAF mutations as both a prognostic and therapeutic marker in Cushing’s disease [33,37,41].

### 3.5. TP53 and ATRX Mutations

The TP53 gene is one of the most well-known tumor suppressors, as it is known to play a central role in maintaining genomic stability by regulating the cell cycle, DNA repair, and apoptosis. The inactivation of this gene through somatic mutations is a hallmark of many aggressive neoplasms, including certain pituitary tumors [42,43,44]. In the context of CD, TP53 mutations, although relatively rare, have been increasingly recognized to play a role, particularly in the more aggressive and invasive corticotropinomas. A study by Perez-Rivas et al. [21] examined 86 functional corticotroph tumors and found pathogenic TP53 mutations in approximately 10% of cases, all of which were macroadenomas and USP8-wild type tumors. Importantly, these TP53-mutant tumors were significantly larger, more invasive (based on Knosp grades), and presented with a higher Ki-67 proliferation index, compared to wild-type tumors. Patients with TP53-mutated corticotroph tumors also required more frequent therapeutic interventions, including third-line strategies such as radiotherapy and bilateral adrenalectomy, suggesting a strong link between TP53 mutations and a more aggressive clinical course [21]. Supporting these observations, another study by Uzilov et al. [45] found that TP53 mutations were strongly associated with an aggressive clinical course. These tumors were predominantly macroadenomas and showed a high rate of invasiveness, with 80% invading the cavernous sinus, and they also presented with a similarly high risk of postoperative recurrence. Additionally, TP53-mutant tumors exhibited a marked genomic instability, characterized by numerous chromosomal alterations and copy number variations, suggesting that TP53 loss disrupts genome stability, promoting the tumor’s progression [45]. In the cohort analyzed by Pękul et al. [46], TP53 mutations were detected in both CD-associated tumors and silent corticotroph tumors, and all were associated with macroadenomas (>10 mm) and invasive growth. Their survival analysis confirmed that TP53-mutant tumors were associated with a worse overall prognosis in CD patients, despite the low mutation frequency. Similarly, other studies have also found TP53-mutated corticotroph tumors to be associated with high Ki-67 indexes and invasive behavior, and a study by Tanizaki et al. [44] also found mutations of this gene in pituitary carcinomas [44,46]. Overall, these findings highlight TP53 mutations as important biomarkers of aggressiveness and a poor prognosis in corticotroph tumors. While rare compared to mutations in USP8 or USP48, TP53 mutations have significant clinical implications, suggesting the need for closer monitoring and potentially more aggressive multimodal management in affected patients [13]. Further research is needed to evaluate whether TP53 status could guide therapeutic decision-making, including the consideration of chemotherapy or targeted agents for TP53-driven tumors.

Similar to TP53, ATRX mutations have also emerged as potential markers of aggressive behavior and a poor prognosis in corticotroph tumors. The ATRX gene encodes a protein involved in chromatin remodeling and telomere maintenance, and its inactivation has been implicated in various high-grade neuroendocrine tumors and central nervous system malignancies [47,48]. A multicenter study by Casar-Borota et al. [49] analyzed a large cohort of aggressive pituitary tumors and pituitary carcinomas, including 22 corticotroph tumors, and found that ATRX loss-of-function mutations were present in 19% of cases, with a higher prevalence among corticotroph tumors (32%) compared to other PitNET subtypes. Importantly, ATRX mutations were more common in pituitary carcinomas (28%) than in aggressive but non-metastatic tumors (13%), supporting the hypothesis that ATRX loss contributes to metastatic potential. Additionally, Sumislawski et al. [50] reported a case of an ACTH-secreting pituitary carcinoma with a metastatic spread to the liver and vertebrae, in which both ATRX and TP53 mutations were identified. Furthermore, the 2022 WHO classification now recognizes ATRX alterations as potential markers of aggressive behavior in PitNETs, especially in corticotroph tumors [47,49]. Overall, the current evidence suggests that ATRX mutations are associated with highly aggressive corticotroph tumors, which have an increased invasiveness, resistance to standard therapies, and metastatic potential.

### 3.6. CABLES1 Mutations

CABLES1 (Cdk5 and Abl enzyme substrate 1) is a cell cycle regulator that plays an important role in controlling cell proliferation and differentiation. It has been widely studied in various types of tumors, where its loss has been associated with uncontrolled tumor growth, impaired cell cycle arrest, and resistance to apoptosis. In several malignancies, such as lung, ovarian, and colorectal cancers, an inactivating mutation in CABLES1 was found to be associated with dysregulated cell cycle progression and increased tumor aggressiveness. Additionally, studies have shown that CABLES1 interacts with tumor suppressors such as p53 and β-catenin, suggesting that its loss may contribute to tumor progression through multiple oncogenic pathways [51]. Given CABLES1 mutational status’s known role in several types of neoplasms, recent research has focused on its relevance in pituitary tumors, including corticotropinomas, where cell cycle dysregulation plays a central role in tumor development [52,53]. In the context of Cushing’s disease, CABLES1 has been identified as an important mediator of glucocorticoid negative feedback on corticotroph cell proliferation. Glucocorticoids normally inhibit corticotroph cell growth by activating cell cycle suppressors, including p27Kip1, a regulator of cell cycle arrest. However, studies have shown that CABLES1 expression is frequently lost or significantly reduced in corticotroph tumors, disrupting this regulatory mechanism [48,52]. In such cases, where the CABLES1 function is lost, glucocorticoid signaling fails to suppress cell proliferation, leading to persistent corticotroph growth and hormone secretion, thus explaining why many corticotroph tumors remain insensitive to glucocorticoid-induced suppression. The genetic basis for CABLES1 disruption in CD was further explored in a study by Hernández-Ramírez et al. [53], who conducted a genetic analysis of 146 pediatric and 35 adult patients with CD. They identified four potentially pathogenic missense mutations in CABLES1 and found that all patients harboring these mutations presented with macroadenomas, suggesting a link between CABLES1 loss and an increased tumor size. The study also proposed that CABLES1 mutations could represent a novel pituitary tumor-predisposing mechanism, particularly affecting interactions with cyclin-dependent kinases (CDKs) and the epidermal growth factor receptor (EGFR) pathway [53]. All these findings highlight the important role of CABLES1 as a pituitary tumors suppressor gene, as its loss may be linked to dysregulated tumor growth, the failure of negative feedback inhibition, and a potentially more aggressive disease progression. Further research is needed to clarify the role of these mutations as a biomarker for CD or even for developing new therapeutic strategies for patients with treatment-resistant or recurrent Cushing’s disease.

In summary, multiple somatic mutations have been identified in corticotroph tumors, with USP8 remaining the most frequently described and clinically relevant to date. Other genetic events such as USP48, USP11, TP53, ATRX, BRAF, and CABLES1 contribute to tumor heterogeneity and may carry prognostic or therapeutic implications, although their routine clinical use remains limited at this stage. A summary of the major genetic alterations identified in corticotroph tumors, along with their clinical significance, is provided in Table 1.

## 4. Immunohistochemical Biomarkers

### 4.1. Granulation Pattern: Densely vs. Sparsely Granulated Corticotroph Tumors

The granulation pattern is a well known and important histopathological feature used to classify corticotroph tumors into densely granulated (DG) and sparsely granulated (SG) subtypes. This distinction is primarily based on an ACTH immunohistochemistry and the distribution of secretory granules within tumor cells, but pathologists often employ cytokeratin staining by CAM 5.2, an antibody that targets low-molecular-weight cytokeratins (CK8/18), alongside periodic acid–Schiff (PAS) staining. In practice, DG tumors show strong ACTH staining and paranuclear dot-like CAM 5.2 positivity, while SG tumors exhibit weaker, patchier ACTH staining with diffuse cytoplasmic CAM 5.2 expression [14,54,55]. The clinical relevance of the granulation pattern has been emphasized by multiple recent studies. In a large retrospective study by Rak et al. [15], which included 277 corticotroph tumors, SG tumors accounted for approximately 20% of cases. These tumors were significantly more likely to exhibit invasiveness, with higher Knosp grades, larger tumor volumes, and a lower chance of achieving postoperative remission compared to the DG tumors. Notably, even after adjusting for tumor size, the granulation pattern itself emerged as an independent prognostic factor for biochemical remission [15]. Similarly, in the study by Durmuş et al. [55], SG tumors were associated with higher mitotic counts, elevated Ki-67 indexes, and a higher T2 intensity on magnetic resonance imaging (MRI) compared to the DG tumors. The authors also observed that SG tumors were more likely to invade the cavernous sinus and were associated with a lower remission rate after surgery and frequently with second-line adjuvant therapies such as radiotherapy [55]. Supporting these observation, another study by Çiftçi Doğansen et al. [54] reported that DG tumors were significantly more likely to be microadenomas and typically responded better to transsphenoidal surgery, while SG tumors were frequently identified as macroadenomas with invasive features [54]. Furthermore, the 2022 WHO Classification of PitNETs now incorporates the granulation pattern as a recommended part of tumor subtyping, highlighting its prognostic utility [14]. Moreover, several studies found that the subtyping by the granulation pattern also has therapeutic significance. In this regard, the study by Castellnou et al. [20] found that SG tumors exhibit a lower expression of somatostatin receptors, potentially explaining their poorer response to Pasireotide and other somatostatin analogs compared to DG tumors [20]. The relationship between granulation pattern and pharmacological treatment response has been well established in somatotropinomas, where DG tumors generally exhibit a higher SSTR2 expression and respond favorably to first-generation somatostatin analogs, whereas SG tumors tend to present a lower SSTR2 and relatively higher SSTR5 expression, making them more responsive to Pasireotide [56,57,58]. However, in corticotroph tumors, the available evidence regarding a similar correlation between granulation pattern and somatostatin receptor expression or treatment response remains limited. Further research is needed to determine whether these patterns observed in somatotropinomas also apply to corticotropinomas and to clarify the predictive value of granulation patterns in guiding medical therapy for Cushing’s disease. In summary, the assessment of the granulation pattern is essential not only for a complete histopathological diagnosis but also for predicting the tumor’s behavior, prognosis, and potentially response to different treatment strategies in Cushing’s disease.

### 4.2. E-Cadherin

E-Cadherin is another IHC biomarker frequently used to assess the aggressiveness of various types of tumors. This cell-adhesion protein is involved in mediating epithelial cell-cell adhesion and maintaining tissue integrity, and it plays a well-established role in inhibiting tumor cell invasion and metastasis. In several tumor types, including neuroendocrine neoplasms, E-cadherin loss or downregulation is associated with epithelial-to-mesenchymal transition (EMT), facilitating cell detachment, migration, and local tissue invasion [22]. In pituitary tumors, particularly somatotropinomas, multiple studies have reported that a reduced E-Cadherin expression is linked to aggressive histological subtypes, correlating with an increased tumor size, invasiveness, and a reduced responsiveness to somatostatin analogs [59,60,61]. In contrast, the role of E-Cadherin in corticotroph tumors is less well established. An early study by Evang et al. [23] investigated E-Cadherin IHC expression in a series of ACTH-secreting pituitary tumors and observed that membranous E-Cadherin expression was well-preserved in most corticotroph microadenomas but progressively decreased in larger and more invasive macroadenomas. Furthermore, the aberrant nuclear localization of E-Cadherin was identified in more aggressive tumors, raising the possibility that E-Cadherin mislocalization may be linked to tumor progression through EMT-like mechanisms [23]. More recent data from a study by Kiseljak-Vassiliades et al. [62] in 2024, however, challenge the prognostic significance of E-Cadherin in corticotroph tumors. In their multicenter cohort, the loss of or a reduction in E-Cadherin expression was commonly observed, but no significant correlation was found between E-Cadherin status and key clinical parameters, such as tumor size, cavernous sinus invasion, recurrence risk, or granulation pattern [62]. In summary, while E-Cadherin loss in corticotropinomas, similar to other subtypes of pituitary tumors, seems to be involved in tumor dedifferentiation and EMT-like changes, its clinical utility as a biomarker for invasiveness or a poor prognosis in Cushing’s disease remains uncertain. Unlike in somatotropinomas, where reduced E-Cadherin is a more consistent marker of aggressive behavior, corticotroph tumors show more variable patterns, and further studies are needed to clarify its significance in this tumor subtype [23,56,59].

### 4.3. Ki-67 Index

The Ki-67 labeling index is a widely used marker of cellular proliferation, reflecting the proportion of tumor cells actively engaged in the cell cycle. The Ki-67 protein is detected using the MIB-1 monoclonal antibody and is considered an important tool for evaluating the biological behavior of pituitary tumors, including corticotropinomas. Although its role in pituitary tumor classification has evolved, Ki-67 continues to be considered as a marker of potential aggressiveness and proliferative activity in clinical practice [24,63]. In corticotroph tumors, several studies have shown that a Ki-67 index ≥3% is suggestive of a more aggressive phenotype, even though this threshold has been debated in recent years. The 2004 WHO classification previously included this marker in the definition of atypical adenomas, but subsequent WHO updates have removed this categorization, while maintaining its clinical utility as a risk indicator when combined with other features such as p53 immunoreactivity [4,14,49,64]. Furthermore, the European Society guideline for the management of aggressive pituitary tumors from 2018 recommends that all pituitary tumors should undergo an evaluation of the Ki-67 index as part of the histopathological analysis [65]. A few recent studies have attempted to evaluate the associations of Ki-67 expression with tumor characteristics such as size, invasiveness, and risk of recurrence in ACTH-secreting pituitary tumors. In a study by Garbicz et al. [64], corticotroph macroadenomas and invasive tumors were found to possess significantly higher Ki-67 indexes compared to non-invasive and smaller tumors, supporting its role as a marker of invasiveness. The results of this study found that invasive corticotroph tumors had a mean Ki-67 of 5.7%, compared to 1.9% in non-invasive tumors [64]. Furthermore, high Ki-67 indexes have been associated with tumor recurrence after surgical treatment. In this regard, a study by Ünal et al. [66] reported that recurrent corticotroph tumors exhibited higher Ki-67 levels compared to those in sustained remission. In this study, 40% of patients with recurrent Cushing’s disease had Ki-67 indices above 3%, while only 25% of patients in sustained remission exhibited similar levels [66]. Another study by Witek et al. [67] prospectively analyzed 59 patients with corticotroph tumors, including both micro- and macroadenomas, and examined the relationship between Ki-67 expression and tumor behavior. The authors found that macroadenomas had significantly higher Ki-67 indexes compared to microadenomas, with Ki-67 expression increasing progressively with tumor size. Interestingly, macroadenomas with a higher Ki-67 were also associated with a more invasive profile, as assessed by the Knosp grading. The study also reported that tumors with Ki-67 indexes ≥3% showed a trend toward higher rates of second surgeries and reduced early remission rates following transsphenoidal surgery, although this did not reach statistical significance [67]. In a larger retrospective study by Rak et al. [15] involving 277 patients with Cushing’s disease, Ki-67 expression was also compared between densely and sparsely granulated corticotroph tumors. The researchers reported that SG tumors were more frequently associated with elevated Ki-67 levels (≥3%) compared to DG, suggesting a link between granulation pattern and proliferative activity [15]. All these results support the theory that increased Ki-67 expression may be linked to a higher likelihood of disease recurrence and serves as useful biomarker for estimating tumor aggressiveness in corticotroph tumors. Despite these observations, the Ki-67 index is not sufficient as a standalone prognostic tool, making a combination with additional molecular and histopathological markers recommended to provide a comprehensive assessment of tumor behavior [4].

### 4.4. Somatostatin Receptor Type 5 (SSTR5)

Somatostatin receptors (SSTRs) are G-protein-coupled receptors that mediate the inhibitory actions of somatostatin on cells expressing these receptors. There are five known SSTR subtypes (SSTR1–SSTR5), each with distinct tissue distributions and signaling pathways. In pituitary tumors, somatostatin receptor expression has important clinical implications, particularly regarding responsiveness to somatostatin analogues, a commonly used pharmacological therapy. While somatotropinomas primarily express both SSTR2 and SSTR5, which mediate their response to first-generation somatostatin analogs or Pasiroetide, the expression profile in corticotroph tumors is more heterogeneous and involves a predominant expression of SSTR5 [20,68,69]. As mentioned, in corticotroph tumors, SSTR5 is the most abundantly expressed receptor subtype, although the co-expression of SSTR2 can also occur but generally at lower levels compared to GH-secreting tumors. This different expression profile of SSTRs is thought to explain the reduced efficacy of first-generation somatostatin analogs in patients with CD. On the other hand, Pasireotide, a somatostatin analog with a high affinity for SSTR5, is a promising therapeutic option for patients with CD that failed primary surgical treatment [1,7,9]. Van der Hoek et al. were among the first to demonstrate that SSTR5 plays a dominant role in regulating ACTH secretion in corticotroph tumor cells, showing that the selective activation of SSTR5 more effectively inhibits ACTH release compared to SSTR2 activation in vitro [70]. Similarly, Hofland et al. reported that Pasireotide, through its SSTR5-mediated mechanism, significantly inhibited ACTH secretion in primary cultures of human corticotroph adenomas, reinforcing the therapeutic relevance of SSTR5 targeting in CD [71]. While several studies have suggested a strong SSTR5 IHC expression might be linked to SG tumors and responsiveness to Pasireotide in GH-secreting pituitary tumors, the data regarding the predictive role of SSTR5 IHC expression in corticotropinomas are less clear [57,72,73]. The study by Castellnou et al. [20] mentioned earlier in this article demonstrated that SSTR5 expression was more frequent in functioning tumors compared to the silent ones and was associated with USP8 mutations. Moreover, their findings confirmed that USP8-mutant tumors tend to exhibit a higher SSTR5 expression, potentially explaining their enhanced responsiveness to Pasireotide therapy, as among the five patients treated with Pasireotide, those with higher IHC scores for SSTR5 generally showed better biochemical responses, although the sample size was too small for definitive conclusions [20]. Additionally, in the study performed by Hayashi et al., SSTR5 expression was significantly higher in USP8-mutant corticotroph tumors, confirming this link between mutational status and somatostatin receptor profile [34]. Overall, all these previously mentioned findings suggest that SSTR5 expression may serve as a useful predictive marker for responsiveness to Pasireotide in corticotroph tumors, particularly in those harboring USP8 mutations. However, further clinical studies are required to validate this association and to explore its potential integration into future personalized treatment algorithms [35].

### 4.5. Dopamine Receptor Type 2 (D2)

Dopamine receptors (DRs), specifically the D2 subtype, play an important role in regulating hormone secretion in the pituitary gland, most notably through the inhibition of the release of prolactin in lactotroph cells. Dopamine receptor agonists, such as cabergoline and bromocriptine, have long been established as the standard therapy for prolactinomas due to their ability to bind to D2 receptors and suppress tumor activity. While the utility of dopamine agonists in other pituitary tumor types is less well defined, emerging evidence has highlighted the relevance of D2 receptor expression in corticotroph tumors as well [74,75,76,77,78,79,80]. Studies evaluating D2 receptor expression in corticotroph tumors have demonstrated variable results, though most reports indicate that functional D2 receptors are present in a majority of these tumors. In a older study by Pivonello et al. [79], D2 receptor expression was detected in approximately 80% of corticotroph tumors. In the same study, tumors expressing D2 receptors exhibited a significant in vitro inhibition of ACTH secretion when treated with dopamine agonists such as cabergoline or bromocriptine. Moreover, in vivo cabergoline treatment over a three-month period resulted in cortisol suppression in 60% of patients, with the normalization of cortisol levels in approximately 40% of cases, but only in D2 receptor-positive tumors [79]. While cabergoline appears to provide a clinical benefit for a subset of patients with corticotroph tumors, the degree of response is highly variable and likely depends on the level of D2 receptor expression on the tumor cells. Unlike prolactinomas, where D2 receptor expression is generally robust and consistent, corticotroph tumors present heterogeneous D2 receptor profiles, which may partially explain the modest efficacy of dopamine agonists in CD compared to their excellent efficacy in prolactinomas [11,77,79]. Despite the evidence supporting the role of D2 receptors as potential therapeutic targets in corticotroph tumors, clinical guidelines still recommend dopamine agonists such as cabergoline primarily as a second- or third-line therapy, particularly in patients who are not candidates for surgery or other pituitary-directed treatments [1,81]. Further prospective studies are required to better understand the potential of D2 expression as a biomarker to guide the therapeutical choice in patients of CD, by predicting the potential responsiveness to dopamine agonists.

### 4.6. Filamin A (FLNA)

Filamin A (FLNA) is a large actin-binding cytoskeletal protein with dual roles in oncogenesis, depending on its subcellular localization (cytoplasmatic or nuclear) and binding partners. FLNA acts both as a structural protein involved in maintaining cytoskeletal integrity and as a scaffold coordinating various signaling pathways. In pituitary tumors, FLNA has been proposed as a potential biomarker for predicting both the response to pharmacological therapy and tumor invasiveness [82,83,84,85]. In the context of CD, FLNA has been identified as a potential modulator of SSTR5’s expression and function, which may have direct implications for the responsiveness to Pasireotide therapy. In a study by Treppiedi et al. [86], FLNA was shown to be essential for maintaining SSTR5 expression and mediating Pasireotide-dependent signaling in corticotroph tumor cells. In their research, it was observed that the silencing of FLNA in both human primary cultures of corticotrophinomas and in murine tumor cells resulted in a significant downregulation of SSTR5 expression, as well as the abolition of Pasireotide-induced antiproliferative and pro-apoptotic effects. In addition, the study revealed that Pasireotide treatment increased the formation of FLNA-SSTR5 complexes, further supporting the idea that FLNA scaffolding is crucial for proper receptor localization and downstream signaling [86]. These findings suggested that FLNA may serve as a predictive biomarker for responsiveness to Pasireotide in CD, although additional clinical studies are required to validate this observation. Furthermore, another study by Sickler et al. [87] investigated FLNA and D2 receptor expression in a series of corticotropinomas. While no correlation between FLNA and D2 expression was found, FLNA expression was associated with tumor invasiveness, notably sphenoidal sinus invasion. These results suggested that FLNA could also be used as a biomarker of tumor aggressiveness in corticotroph tumors, beyond its role in receptor signaling modulation [87]. Earlier studies in other PitNET subtypes, such as prolactinomas, also revealed FLNA’s role in modulating D2 signaling. In lactotroph tumors, a reduced FLNA expression was linked to dopamine agonist resistance due to an impaired D2 membrane expression and function, as demonstrated by another study by Peverelli et al. [88]. However, the role of FLNA in predicting dopamine agonists’ response in CD remains to be established in future studies. In summary, FLNA emerges as a novel molecular marker for various tumor types, including corticotropinomas, due to its involvement in receptor dynamics and signaling tumoral cells. FLNA was shown to have regulatory effects on SSTR5, thus making it a promising predictive marker for responsiveness to Pasireotide in CD, while its correlation with invasive behavior might help in predicting tumors’ behavior and in prognosis. Further prospective studies are necessary to establish its clinical utility as a biomarker.

In summary, several immunohistochemical markers have been proposed for the evaluation of corticotroph tumors, ranging from Ki-67 proliferation indexes to receptor expression patterns. While none of the markers described in this review are currently sufficient as standalone prognostic or predictive tools, their combined interpretation may support treatment selection and outcome prediction, and future management algorithms may integrate these markers to facilitate a personalized approach. The main immunohistochemical biomarkers investigated in corticotroph tumors, along with their clinical relevance, are summarized in Table 2.

## 5. Conclusions

In conclusion, recent advances in the molecular characterization of corticotroph tumors have identified several biomarkers with potential clinical relevance in the management of Cushing’s disease. Among these, USP8 mutations remain the most extensively studied genetic alteration, being present in up to 60% of corticotropinomas and associated with microadenomas, lower rates of invasiveness, and higher remission rates after surgery. Furthermore, tumors with a USP8 mutation show increased SSTR5 expression, which may correlate with an improved biochemical response to Pasireotide, suggesting that USP8 status may serve as a predictive biomarker for responsiveness to this pharmacological agent. In contrast, the TP53 and ATRX mutations, although less frequent, are consistently linked to larger, invasive macroadenomas with high Ki-67 indexes, a poor response to first-line treatments, and a higher risk of recurrence or aggressive behavior. These biomarkers may help identify patients who require closer postoperative surveillance or the consideration of earlier adjuvant, second-line therapies. USP48 mutations are found in a subset of USP8-wild-type tumors and are associated with a smaller tumor size and preserved feedback responsiveness to suppressive factors, but their clinical implications are still unclear. USP11 seems to emerge as a key driver of sex-biased ACTH overproduction in Cushing’s disease and a promising target for therapeutic intervention. BRAF mutations, though rare and predominantly reported in East Asian populations, activate MAPK signaling and could represent a future therapeutic target in select patients. Regarding CABLES1, inactivating mutations have been reported in corticotropinomas, especially larger macroadenomas and predominantly in pediatric or early-onset cases, although these mutations may represent a tumor predisposition mechanism rather than a direct driver of resistance to therapy, making their role as a predictive biomarker uncertain. Despite this, their identification could be relevant in selected patients with early-onset or familial forms of CD.

Among the molecular markers evaluated through IHC, a sparsely granulated pattern, Ki-67 index ≥3%, and reduced E-Cadherin expression have been linked to enhanced invasiveness, lower surgical remission rates, and a risk of recurrence in corticotropinomas. The IHC expression of SSTR5 and D2 receptors may help in deciding pharmacological treatment choices, especially when considering Pasireotide or cabergoline, which target these two receptors. Despite this, the routine application of IHC for these markers in clinical practice remains limited due to a lack of standardized thresholds and insufficient evidence.

While all these available data about the roles of molecular biomarkers are encouraging, most of them are not yet routinely integrated into clinical protocols and management algorithms due to a variability in methodologies, cutoff values, and study designs. Well-designed, prospective studies with larger patient cohorts are needed to validate their predictive value and to define how they can be used in future guidelines. In the future, the integration of validated genetic and immunohistochemical biomarkers into clinical practice may allow for a personalized therapeutic approach for patients with CD, with the ultimate aim of defining an accurate prognosis and improving long-term outcomes for patients with this challenging disorder.

One limitation of our study is the exclusion of methylation regulatory networks and immune microenvironment integration, as our emphasis was on identifying biomarkers that are more practical and accessible for current assessments.

**Figure 1:**
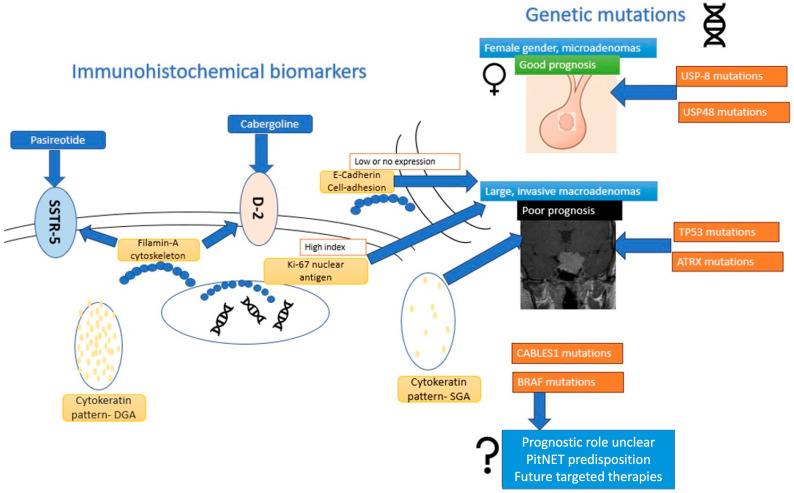
Schematic representation of main IHC biomarkers and genetic mutations in CD—clinical implications, prognosis, and pharmacological treatment predictive value.

A visual overview of the main genetic and immunohistochemical biomarkers in corticotroph tumors discussed in this article, including their clinical implications and implications in pharmacological therapy for CD, is provided in Figure 1**.**

## Figures and Tables

**Table 1:** Summary of genetic alterations in corticotroph tumors and their clinical implications.

Gene	Prevalence	Associations	Clinical Implications	References
**USP8**	30–60%	Microadenomas, female predominance, less invasiveness	Higher remission rates post-surgery, ↑ SSTR5 expression, potential predictive marker for Pasireotide response	[25,26,27,28,29,30,31,32,33,34,35,36]
**USP4**	10–23% (in USP8-wild-type tumors)	Smaller tumors, female tendency	Potential role in ACTH overproduction, CRH sensitivity, therapeutic target in USP8-WT tumors	[37,38,39]
**USP11**	-	Female predominance	A promising therapeutic target for novel treatments	[40]
**BRAF**	~10–16% (mostly Asian cohorts)	↑POMC/ACTH secretion	Rare; possible target for MAPK/ERK inhibitors	[33,37,39,41]
**TP53**	~10%	Macroadenomas, ↑Ki-67, cavernous sinus invasion	Aggressive behavior, recurrence, poor prognosis, invasive growth	[21,42,43,44,45,46]
**ATRX**	~19–32% (in aggressive forms)	Carcinomas or highly aggressive tumors	Associated with metastasis and resistance to therapy	[47,48,49]
**CABLES1**	Rare (mainly pediatric macroadenomas)	Macroadenomas, loss of feedback inhibition	May explain glucocorticoid resistance; potential tumor suppressor marker	[48,51,52,53]

**Table 2:** IHC biomarkers and their clinical relevance in corticotroph tumors.

Biomarker	Function	Clinical Implications	References
**SSTR5**	Somatostatin receptor subtype 5—target of somatostatin ligands	↑ in USP8-mutated tumors → better response to Pasireotide	[20,33,70,71,72,73]
**D2**	Dopamine D2 receptor—target of dopamine agonists	Variably expressed; may predict responsiveness to Cabergoline	[79,80,81]
**FLNA**	Cytoskeleton actin-binding scaffolding protein	Stabilizes SSTR5; may be involved in Pasireotide signaling and response;some data support associations with invasiveness	[86,87,88]
**Ki-67**	Cell proliferation index	↑ in aggressive tumors (TP53, SG subtype); associated with recurrence, invasiveness	[63,64,66,67]
**E-Cadherin**	Cell adhesion molecule	↓ linked with tumor dedifferentiation and invasiveness, poor prognosis	[23,56,59,60,61]
**Granulation pattern**	Secretory granule density pattern	SG subtype = larger, invasive tumors, worse prognosis	[14,15,54,55,56,57,58]

↑ = higher expression; ↓ = lower expression.

## Abbreviations

The following abbreviations are used in this manuscript:

CD	Cushing’s disease
ACTH	Adrenocorticotropic hormone
USP8	Ubiquitin-specific protease 8
CABLES1	Cdk5 and Abl enzyme substrate 1
SSTR5	Somatostatin receptor subtype 5
WHO	World Health Organization
EGFR	Epidermal growth factor receptor
USP48	Ubiquitin-specific protease 48
IHC	Immunohistochemistry
POMC	Pro-opiomelanocortin
CRH	Corticotropin-releasing hormone
MGMT	Methylguanine-DNA–methyltransferase
MAPK	Mitogen-activated protein kinase
DG	Densely granulated
SG	Sparsely granulated
PAS	Periodic acid–Schiff
MRI	Magnetic resonance imaging
EMT	Epithelial–mesenchymal transition
GH	Growth hormone
D2	Dopamine receptor D2
FLNA	Filamin A
